# ATM–Dependent MiR-335 Targets CtIP and Modulates the DNA Damage Response

**DOI:** 10.1371/journal.pgen.1003505

**Published:** 2013-05-16

**Authors:** Nathan T. Martin, Kotoka Nakamura, Robert Davies, Shareef A. Nahas, Christina Brown, Rashmi Tunuguntla, Richard A. Gatti, Hailiang Hu

**Affiliations:** 1 Department of Pathology and Laboratory Medicine, University of California Los Angeles, Los Angeles, California, United States of America; 2 UCLA Biomedical Physics Interdepartmental Graduate Program, University of California Los Angeles, Los Angeles, California, United States of America; 3 Jonsson Comprehensive Cancer Center, University of California Los Angeles, Los Angeles, California, United States of America; 4 Department of Human Genetics, David Geffen School of Medicine, University of California Los Angeles, Los Angeles, California, United States of America; Memorial Sloan-Kettering Cancer Center, United States of America

## Abstract

ATM plays a critical role in cellular responses to DNA double-strand breaks (DSBs). We describe a new ATM–mediated DSB–induced DNA damage response pathway involving microRNA (miRNA): irradiation (IR)-induced DSBs activate ATM, which leads to the downregulation of miR-335, a miRNA that targets CtIP, which is an important trigger of DNA end resection in homologous recombination repair (HRR). We demonstrate that CREB is responsible for a large portion of miR-335 expression by binding to the promoter region of miR-335. CREB binding is greatly reduced after IR, corroborating with previous studies that IR-activated ATM phosphorylates CREB to reduce its transcription activity. Overexpression of miR-335 in HeLa cells resulted in reduced CtIP levels and post-IR colony survival and BRCA1 foci formation. Further, in two patient-derived lymphoblastoid cell lines with decreased post-IR colony survival, a “radiosensitive” phenotype, we demonstrated elevated miR-335 expression, reduced CtIP levels, and reduced BRCA1 foci formation. Colony survival, BRCA1 foci, and CtIP levels were partially rescued by miRNA antisense AMO-miR-335 treatment. Taken together, these findings strongly suggest that an ATM–dependent CREB–miR-335–CtIP axis influences the selection of HRR for repair of certain DSB lesions.

## Author Summary

ATM (Ataxia-Telangiectasia Mutated) serine/threonine kinase plays a critical role in coordinating the cellular response to DNA double-strand breaks (DSBs), such as cell cycle checkpoint, DNA repair, and apoptosis. miRNAs have been reported to be involved in many cellular processes, and their role in DSB–triggered DNA damage response (DDR) is just being elucidated. Here we describe a novel DSB response mechanism whereby ATM–dependent miR-335 downregulates CtIP. CtIP is a multifunctional protein that is crucial for DNA homologous recombination repair; and we show, for the first time, that this protein is subject to miRNA downregulation. CtIP downregulation was verified in cell lines from radiosensitive patients, and we demonstrate that this pathway contributes to radiosensitization and DNA repair defects in BRCA1 foci formation and cell cycle checkpoint. Given that miR-335 is also a tumor metastasis suppressor, our finding suggests that miR-335 overexpression could not only increase tumor sensitivity to radiation or chemical therapy but also inhibit tumor metastasis or re-initiation of tumor growth.

## Introduction

The DNA damage response (DDR) plays an essential role in deciding cell fate after DNA double strand breaks (DSBs) by arresting the cell cycle to allow evaluation of DNA integrity and signaling for repair or apoptosis [1]. In this way, the DDR maintains genomic stability and is an indispensable defense mechanism against cell death or tumor development [2], [3]. The molecular mechanisms of DSB-induced DDR have been extensively characterized and post-translational modifications of proteins, such as by phosphorylation, ubiquitinylation, sumoylation and acetylation, play a crucial role [4]. MiRNAs have recently emerged as endogenous gene regulators but their role in the DDR remains largely unexplored. MiRNAs downregulate protein expression by mRNA cleavage or translation repression [5], suggesting that miRNAs may be a new class of cellular regulators, targeting the protein components of the DDR pathways [6].

ATM (Ataxia-Telangiectasia Mutated) kinase plays a hierarchical role in the DSB-induced DDR [7], [8]. ATM coordinates many cellular processes of the ionizing radiation induced DDR starting with the phosphorylation of specific serines or threonines on downstream protein substrates [8], [9]. Three mechanisms have been reported to regulate ATM expression: 1) promoter methylation [10], [11], 2) transcription activators [12] and 3) miRNA interactions with mRNA [13]–[15]. Such modulation of ATM protein levels can result in the radiosensitization of cells [11], [16]. Classically, radiosensitization has been associated with mutations in the *ATM* gene that lead to loss of the protein and failure to activate downstream substrates. Recently, we have shown that ATM protein levels are reduced by miR-421 overexpression and that this reduction in ATM levels is sufficient to induce a cellular phenotype similar to that found in A-T patients [13]. The miR-421-mediated ATM downregulation was recently identified as a major factor associated with clinical radiosensitivity in a patient with squamous cell carcinoma [17]. Interestingly, the ATM/miRNA interaction is bidirectional. In addition to phosphorylating downstream protein targets, ATM also regulates the DDR by modulating the biogenesis of a subset of miRNAs by phosphorylating KH-type splicing regulatory protein (KSRP) which increases mature transcripts of a number of miRNAs [18]. Similarly, the breast cancer susceptibility gene, *BRCA1*, has been shown to modulate miRNA biogenesis [19]. BRCA1 plays an important role in avoiding tumor genesis likely through the maintenance of genomic stability by its role in cell cycle regulation and localization to DSBs thereby facilitating DNA repair [20]–[22]. Thus, ATM and BRCA1 highlight an intriguing connection between miRNA and the DDR.

We have extensively profiled over 100 cell lines derived from patients with A-T-like phenotypes, e.g. reduced clonogenic survival levels characteristic of radiosensitive patients, but of primarily unknown etiology [23], [24]. These ‘radiosensitive’ lymphoblastoid cell lines (RS-LCLs) have been used to interrogate some of the mechanisms of DNA repair [25]–[28]. To complement the methodologies used in previous studies, we performed miRNA microarray measurements on RS-LCLs to identify miRNAs that associate with radiosensitivity and, thus, with DDR defects. In the present study we focus on one of these miRNAs, miR-335, which has been associated with increased metastatic and re-initiation potential of breast cancer when downregulated [29], [30]. MiRNA-335 is located within the mesoderm specific transcript homolog (*MEST*) gene on chromosome 7 which is a maternally imprinted gene [31].

**Figure 1:**
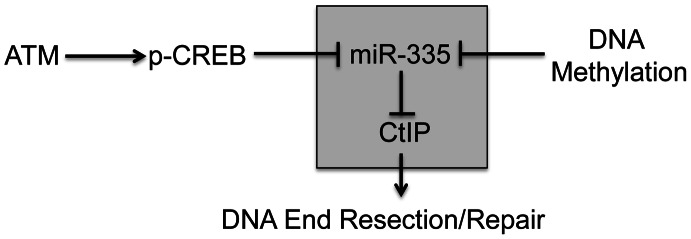
Operational model of ATM–dependent miR-335 DDR modulation pathway. In response to IR, miR-335 is downregulated by pCREB in an ATM dependent manner. MiR-335 downregulates CtIP and likely modulates the initiation of DNA end resection and repair. MiR-335 is also subject to epigenetic regulation by DNA methylation. The shaded box indicates that the miR-335-CtIP signaling axis is modulated by both irradiation and DNA methylation.

We describe a new pathway triggered by ATM-dependent downregulation of miR-335 expression. We show that CREB (cAMP responsive element binding protein 1) binds to the *MEST* promoter and regulates miR-335 in an irradiation (IR)-dependent manner. Further, we demonstrate that this new pathway modulates the DDR via miR-335 suppression of CtIP (CtBP interacting protein/RBBP8), a protein that plays an important role in DNA end resection leading to the recruitment of BRCA1 to DSBs during homologous recombination repair (HRR) (Figure 1) [32]. We demonstrate dysfunction of these DDR mechanisms in two patient-derived RS-LCLs with constitutive miR-335 overexpression and rescue their radiosensitive cellular phenotypes by suppressing miR-335.

## Results

### Post-IR ATM phosphorylation of CREB down-regulates miR-335

**Figure 2:**
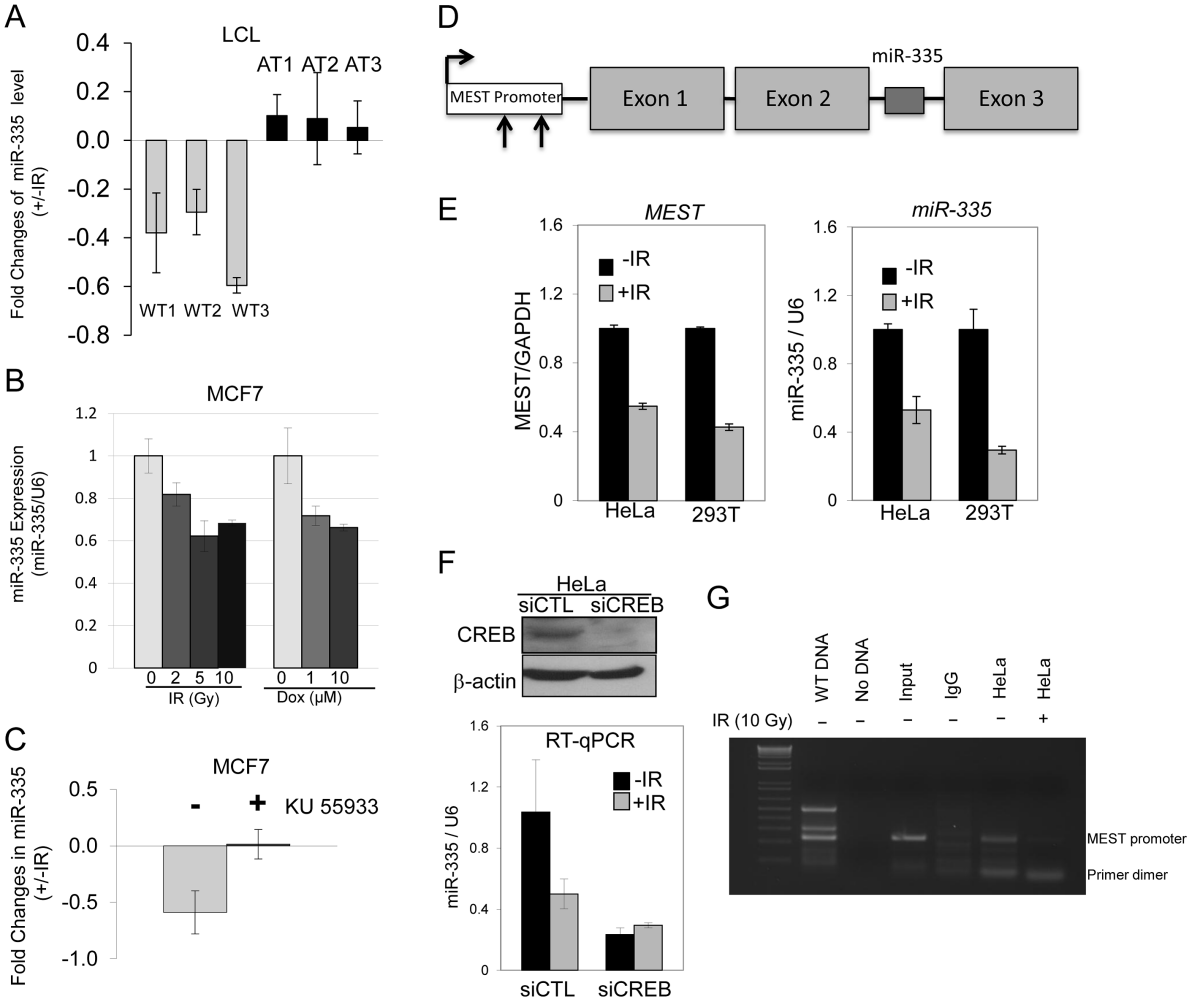
ATM–dependent downregulation of miR-335 post-IR. (A) RT-qPCR indicates downregulation of miR-335 expression 2 hours after 10 Gy in WT but not A-T LCLs. (B) MiR-335 downregulation following IR- or doxorubicin-induced DNA damage by RT-qPCR in MCF7 (ATM +/+) cells. (C) Inhibition of ATM kinase activity in MCF7 cells with KU-55933 for 2 hours prior to IR exposure abolishes the downregulation of miR-335 expression 2 hours after IR. (D) MiR-335 is located in the second intron of *MEST*. The arrows indicate two predicted CREB binding sites in the *MEST* promoter (−938 to −949, −214 to −216 related to translational start site, respectively). (E) RT-qPCR shows that transcription levels of *MEST* mimic those of miR-335 in response to IR, supporting their anticipated co-regulation. (F) Suppression of CREB by siRNA reduced endogenous miR-335 levels and abolished the post-IR down-regulation of miR-335 observed in siCTL transfected HeLa cells. (G) Results of CREB ChIP assay 2 hours post-IR in HeLa cells indicated the binding of CREB to the *MEST* promoter before IR and release from the promoter after IR.

A miRNA microarray screen identified several miRNAs that appeared to have expression levels regulated in an ATM-dependent manner. We chose to focus on miR-335 as it appeared to be downregulated in response to IR in WT-LCLs but not in A-T LCLs. Real time quantitative PCR (RT-qPCR) confirmed that miR-335 was downregulated in all three WT-LCLs post-IR and that this downregulation was absent in the A-T LCLs tested (Figure 2A); a result also found by Smirnov and Cheung [33]. Downregulation of miR-335 post-IR was also true in another ATM proficient cell type, MCF7 cells. Treatment of cells with IR or doxorubicin, another potent DSB inducer, resulted in a similar downregulation of miR-335, indicating that this was likely a general response to DSBs (Figure 2B). The lack of miR-335 downregulation in A-T LCLs strongly suggested that ATM kinase activity is required for the IR-induced miR-335 downregulation observed in WT-LCLs and MCF7 cells. Pre-treatment of MCF7 cells with KU-55933, an ATM kinase specific inhibitor [34], led to the loss of IR-induced miR-335 downregulation (Figure 2C), confirming the requirement of ATM kinase activity for the differential expression patterns observed in WT and A-T LCLs.

Because ATM has not been reported to function as a transcription factor, it was unlikely that it acted directly to repress miR-335 transcription after DNA damage induced by IR. MiR-335 is located within the second (2nd) intron of the *MEST* gene suggesting that miR-335 is co-regulated via a common promoter with its host gene (Figure 2D) [35]. Post-IR damage to HeLa and HEK-293T cells showed downregulation of both miR-335 and *MEST* mRNA by RT-qPCR, suggesting that they are, indeed, co-transcribed under the same promoter (Figure 2E). To identify potential transcription regulatory elements of both *MEST* and miR-335, we analyzed the 2 kb DNA sequence upstream of the translation start site of *MEST* using the transcription factor search program, ConSite. We focused our search on transcription factors known to interact with ATM that might result in the downregulation of miR-335 levels. Two putative CREB binding sites were identified from this search (CACGTCGCGCTG and CGCGGCAACCAG) (Figure 2D). Previous studies have reported that ATM phosphorylates CREB in response to DNA damage and that this phosphorylation reduces its transcription activity [36]. Thus, CREB was postulated to be the likely mechanism for ATM-dependent downregulation of miR-335. We tested this using siRNA to knock down CREB protein expression and found that, indeed, the IR-induced downregulation of miR-335 was abolished in siCREB-transfected HeLa cells (Figure 2F). In addition, pre-IR basal levels of miR-335 were markedly decreased indicating that CREB drives a portion of the endogenous miR-335 expression observed in WT cells (Figure 2F). We used a chromatin immunoprecipitation (ChIP) assay to demonstrate that CREB binds to the *MEST* promoter region in non-irradiated HeLa cells while this binding disappeared post-IR (Figure 2G), suggesting that IR dissociates CREB from the *MEST* promoter, therefore, reducing its transcription effect.

*MEST* is a maternal imprinted gene and the maternal transcript is silenced by DNA methylation. Promoter hypermethylation has previously been shown to regulate *MEST* expression [31]. Treatment of cells with the de-methylation reagent, 5′-azacytidine, indeed revealed that both *MEST* and miR-335 expression levels were significantly up-regulated (Figure S1A), as previously reported [31]. This result supports a working model that *MEST* and miR-335 are co-transcribed and suggests that post-IR phosphorylation of CREB by ATM kinase activity may mediate the downregulation of the paternal transcripts of miR-335 (Figure 1). We confirmed this by monitoring the methylation status of the *MEST* promoter region before and after IR. IR induced no significant change in promoter methylation status (Figure S1B and S1C).

### CtIP protein levels are reduced by miR-335

**Figure 3:**
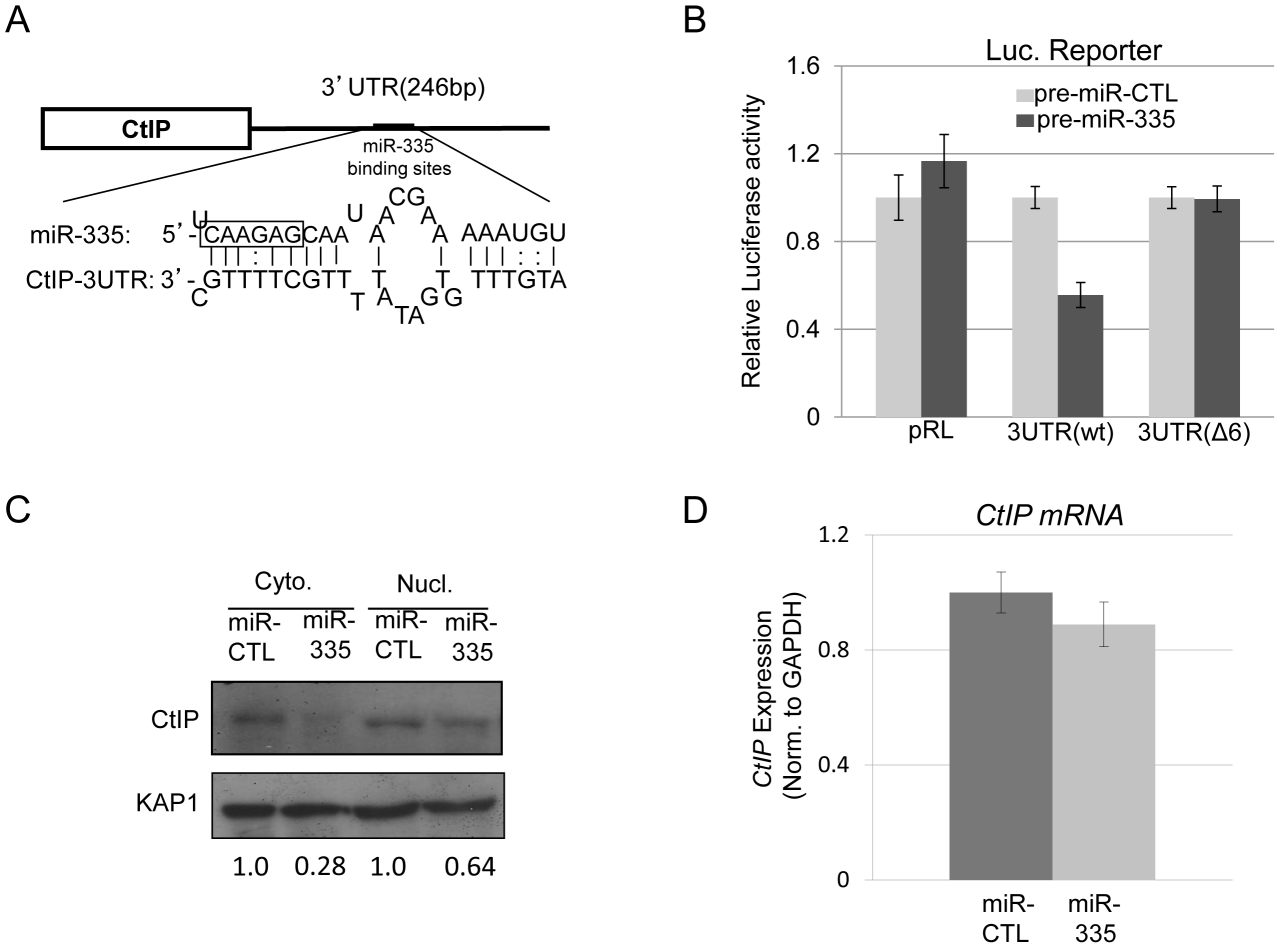
CtIP is a protein target of miR-335. (A) Diagram illustrating the putative miR-335 binding site in the 3′UTR of *CtIP*. The miR-335 seed sequence is boxed. (B) *CtIP* 3′UTR luciferase reporter assay indicated downregulation of luciferase activity after overexpression of miR-335 in HeLa cells using luciferase reporter constructs containing the WT 3′UTR of the *CtIP* gene. This downregulation in luciferase activity was not observed with the reporter construct containing a mutated version of the 3′UTR of CtIP with a deletion of 6-nucleotide miR-335 ‘seed’ sequence (Δ6). (C) Immunoblot of nuclear and cytoplasmic lysates from HeLa cells indicating reductions in CtIP protein levels after overexpression of miR-335. The fold change of CtIP is shown below the blot after normalization to KAP1, a loading control. (D) RT-qPCR results of *CtIP* transcript levels with miR-CTL and miR-335 overexpression in HeLa cells. Expression levels were normalized to *GAPDH* expression as an internal control.

The ATM-kinase dependent downregulation of miR-335 after IR suggested that there might be a link between miR-335 and the DDR. *In silico* analysis of miR-335 by the MicroCosm Target program implicated CtIP, a protein central to HRR, as a target of miR-335 through a putative binding site in its 3′ UTR (Figure 3A and Table S1). To validate the MicroCosm Target prediction, luciferase reporter constructs containing the 3′UTR of CtIP, or variations of this 3′UTR, were generated and co-transfected into HeLa cells with pre-miR-335. Successful overexpression of miR-335 by transfection of pre-miR-335 in HeLa cells was confirmed by RT-qPCR (Figure S2). A significant reduction in luciferase activity was observed after miR-335 overexpression in HeLa cells with the WT 3′UTR-CtIP reporter construct while no changes in activity were observed for the parental, non-specific construct (pRL) (Figure 3B). Further, deletion of the six-nucleotide miR-335 target “seed” sequence (Δ6) disrupted the miR-335-mediated reduction in luciferase activity (Figure 3B). This prompted us to measure endogenous CtIP protein levels in HeLa cells after overexpression of miR-335. MiR-335 overexpression downregulated CtIP protein expression in HeLa cells in both cytoplasmic and nuclear fractions (Figure 3C). Similarly, miR-335 upregulation induced by de-methylation of the *MEST* promoter with 5′-azacytidine resulted in reduced CtIP protein levels (Figure S3). *CtIP* mRNA levels were also assayed after overexpression of miR-335 in HeLa cells and remained unaffected (Figure 3D), supporting the working model that miR-335 directly targets CtIP protein expression and is the most likely link to the DDR in our putative ATM-CREB-miR-335 pathway.

### DNA repair, intra S-phase checkpoints, and radiosensitivity are modulated by miR-335 reduction of CtIP

**Figure 4:**
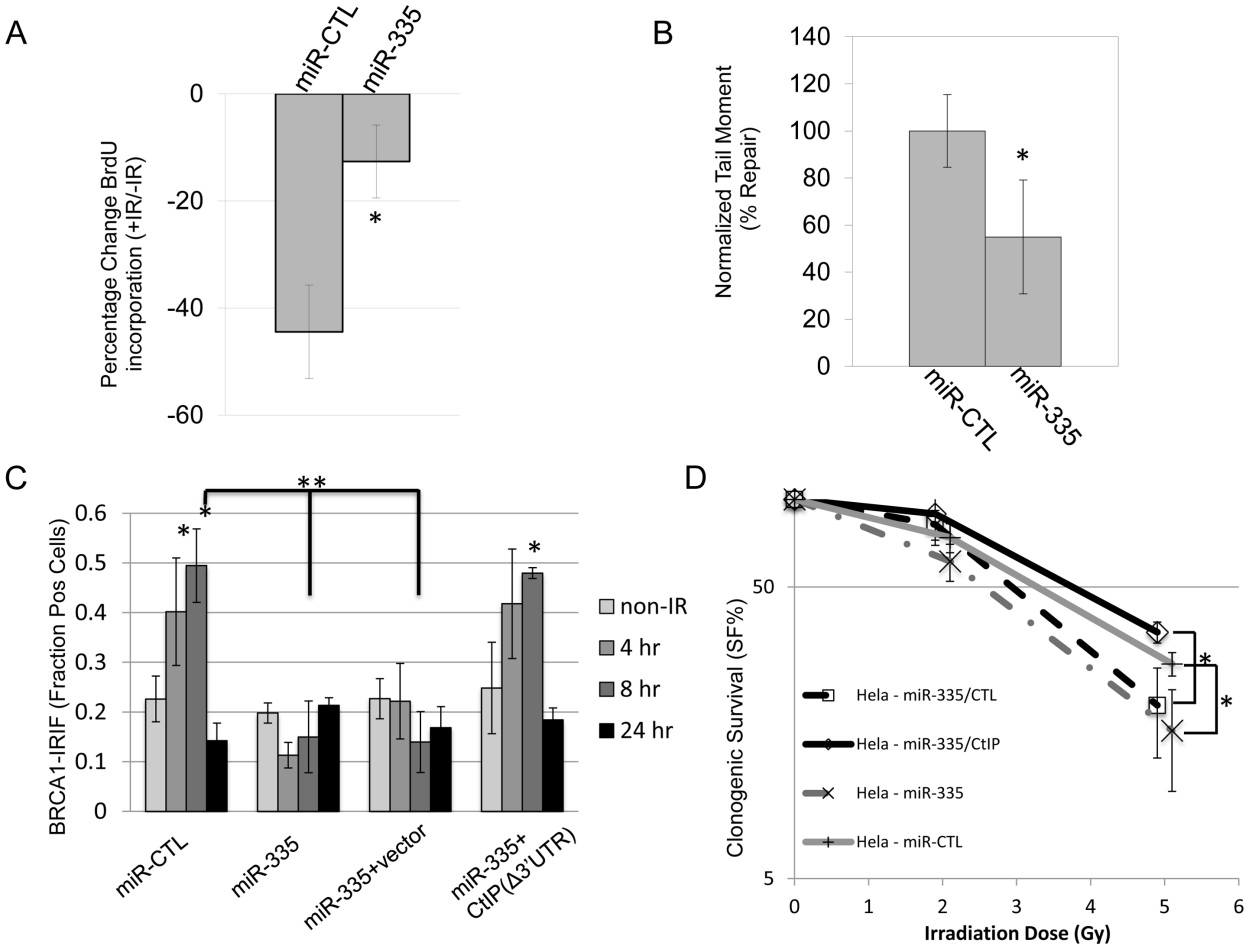
DDR defects induced by miR-335 overexpression in HeLa cells. (A) RDS (radioresistant DNA synthesis): miR-CTL and miR-335 overexpressing HeLa cells were treated with or without 10 Gy IR and S-phase DNA synthesis was labeled with BrdU. The percent change of BrdU incorporation (+IR/−IR) was summarized from three independent experiments. MiR-CTL overexpressing HeLa cells showed a reduction (42%) in the percentage of BrdU incorporation whereas miR-335 overexpressing HeLa cells showed less of a reduction (16%). The * indicates p<0.05. (B) NCA (neutral comet assay): quantification of DNA repair with the NCA indicates reduced DNA repair at 5 hours after 15 Gy in HeLa cells overexpressing miR-335. Three independent experiments were performed; the * indicates p<0.05. (C) BRCA1 foci: immunofluorescent staining of HeLa cells after 12 Gy IR with BRCA1 showed a foci-forming defect in miR-335 overexpressing cells (bars 7 and 11). This response was reversed and corrected in miR-335 overexpressing cells co-transfected with CtIP lacking the 3′UTR (Δ3′UTR) (bar 15). The * indicates intra-sample Student's t-test (p<0.05) comparing the indicated bar with the non-IR condition. ** indicates inter-sample Student's t-test (p<0.05) comparing the 8 hour time points between samples. (D) CSA (clonogenic survival assay): reduced colony survival in miR-335 overexpressing HeLa cells post-IR. The survival fraction was significantly improved when CtIP (Δ3′UTR) was added back to miR-335 overexpressing HeLa cells. The radiation dose used for all samples is 0, 2, and 5 Gy. The x-axis has been offset for each pair of data points to make viewing the data and error bars easier. The * indicates p<0.05.

CtIP regulates DNA end resection during HRR and influences the choice of DNA repair pathway after DSBs [32], [37], [38]. Thus, we hypothesized that the DDR may be modulated by ATM-dependent regulation of miR-335 through resulting changes in CtIP protein levels. Radiation resistant DNA synthesis (RDS) is a characteristic of A-T LCLs, which have a defective intra-S phase cell cycle arrest when responding to IR; a checkpoint CtIP also plays a role in [38]–[40]. An RDS phenotype was induced in HeLa cells by overexpressing miR-335 (Figure 4A and Figure S4). Further, overexpression of miR-335 in HeLa cells induced a delay in DNA repair visualized by residual comet tails 5 hours post-IR in the neutral comet assay (Figure 4B and Figure S5).

BRCA1 interacts with CtIP to facilitate DNA repair [37], [41]. Assessment of BRCA1 foci formation at DNA breaks after IR in HeLa cells demonstrated that miR-335 overexpression reduced BRCA1 foci formation or retention at DSBs and this defect was rescued by transfecting CtIP without the 3′ UTR in to the cells, further supporting the working model that miR-335 modulates the DDR through CtIP (Figure 4C). These DDR defects caused by miR-335 overexpression in HeLa cells suggest that miR-335 may sensitize cells to IR. We observed reduced clonogenic survival when miR-335 was overexpressed in HeLa cells, a trend that was most notable at 5 Gy, indicating that the DDR defects induced by overexpression of miR-335 culminate in sensitization of cells to IR. Re-expression of CtIP without the 3′ UTR was able to de-sensitize the miR-335-overexpressing HeLa cells to IR (Figure 4D).

### ATM-CREB-miR335-CtIP axis was disrupted in two RS-LCLs

**Figure 5:**
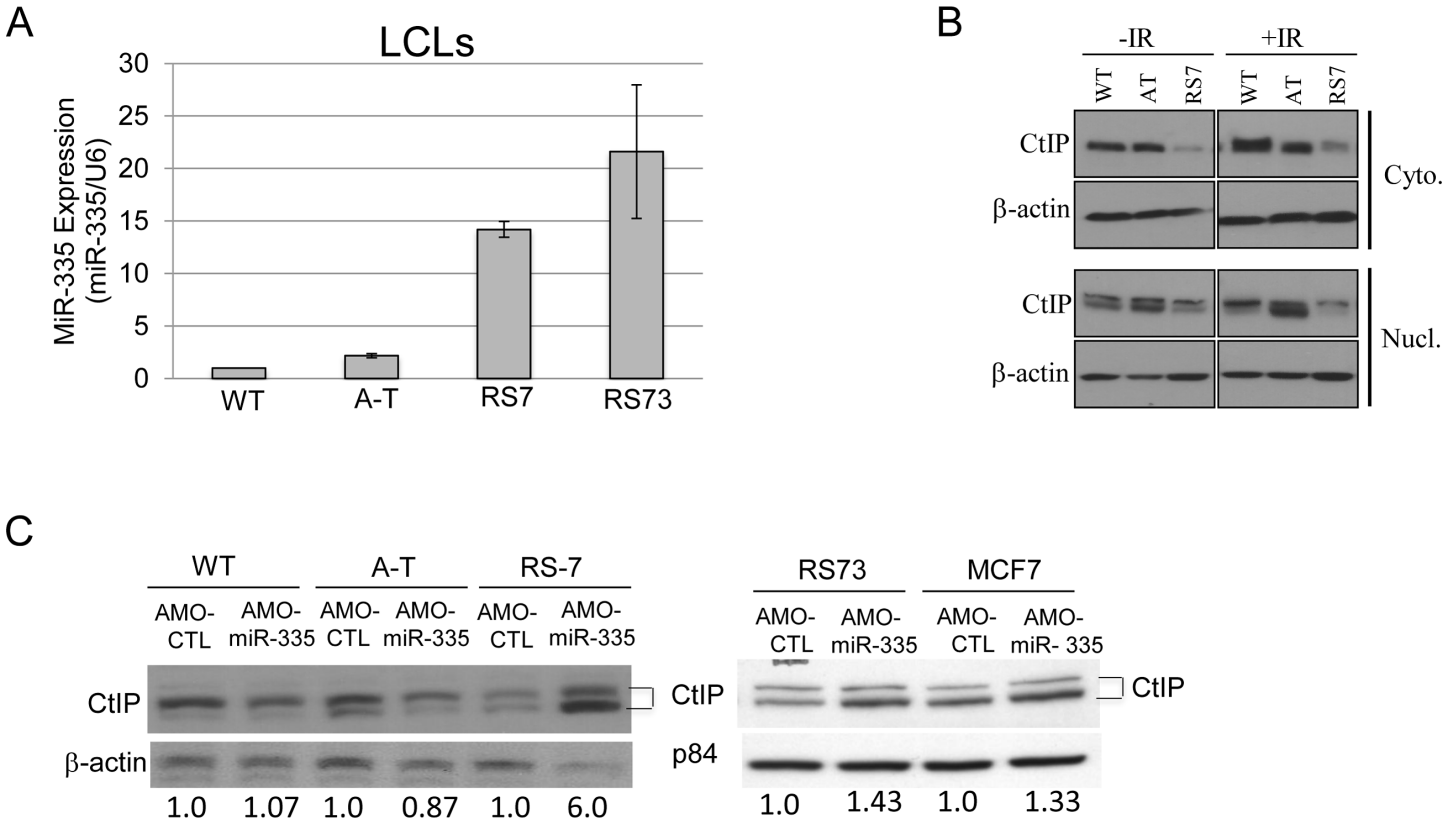
MiR-335 was constitutively overexpressed in RS7 and RS73 LCLs. (A) RT-qPCR indicating >10-fold increases in miR-335 expression in RS7 and RS73 LCLs when compared to WT and A-T LCL controls. Three different WT LCLs were used in RT-qPCR experiments and were used to normalize the expression values for A-T, RS7, and RS73 LCLs. (B) Immunoblotting of cytoplasmic and nuclear lysates isolated from RS7 cells with or without 10 Gy IR indicated reductions in CtIP protein levels. Two bands were noted, representing modified and unmodified CtIP. (C) Treatment of RS7, RS73 and MCF7 cells (also miR-335 overexpressing) with AMO-miR-335 increased nuclear CtIP protein levels compared to WT and AT cells. The quantification of CtIP is shown below the blot after normalization to the loading control.

The miRNA microarrays identified several patient-derived RS-LCLs with elevated levels of miR-335 before and after IR. To further support our working model of the proposed ATM-miR335-CtIP pathway and the impacts of miR-335 expression on the DDR, we chose to focus on two RS-LCLs previously described by our lab, RS7 [25] and RS73 [28], with constitutive miR-335 overexpression. MiR-335 overexpression was validated by RT-qPCR and miR-335 levels were elevated >10 fold when compared to WT and A-T LCL controls (Figure 5A). Significant reduction of CtIP protein expression was observed in RS7 (Figure 5B), similar to HeLa cells overexpressing miR-335 (Figure 3C). To further demonstrate the inverse relationship between miR-335 and CtIP protein levels in the naturally miR-335 overexpressing RS-LCLs, an antisense morpholino oligonucleotide (AMO-miR-335) was used to suppress miR-335 expression. AMO-miR-335 was designed to be complementary to miR-335 thereby inhibiting the processing of pre-miR-335 into mature transcripts and also to block the binding of mature transcripts to mRNA targets. Treatment of RS7 and RS73 LCLs with AMO-miR-335 resulted in increased CtIP protein levels, confirming that overexpression of miR-335 was responsible for reductions in CtIP in both RS7 and RS73 LCLs (Figure 5C).

### MiR-335 disruption of CtIP results in BRCA1 foci defects and “radiosensitivity” in RS7 and RS73 LCLs

**Figure 6:**
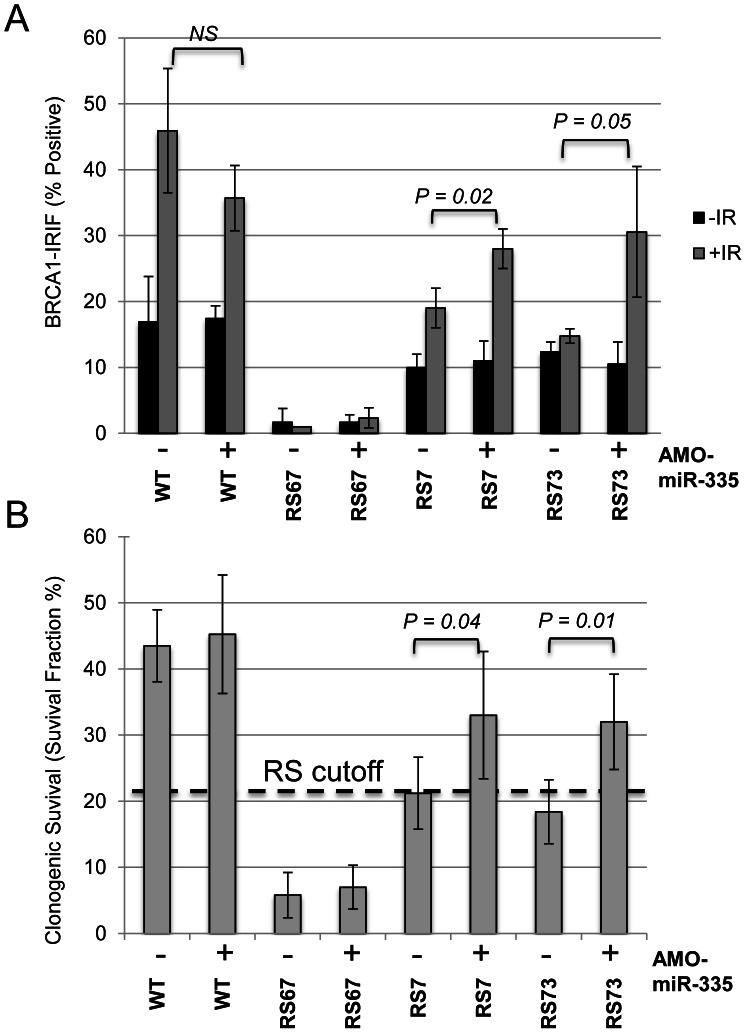
MiR335-induced DDR defects were abrogated in RS7 and RS73 after AMO-miR-335 treatment. (A) Partial restoration of BRCA1-IRIFs 8 hours post-12 Gy in constitutively miR-335 overexpressing RS7 and RS73 LCLs following AMO-miR-335 exposure (+). The (−) denotes similar treatment with AMO-CTL. RS67 is deficient for RNF168 and does not form stable BRCA1-IRIFs [27]; it was used as a negative control for BRCA1-IRIFs in these experiments. (B) Treatment with AMO-miR-335(+) partially corrected the reduced colony survival, a ‘radiosensitive’ cellular phenotype, after 1 Gy IR in RS7 and RS73. The RS cutoff is based on previous optimizations [23].

In a previous study, RS7 cells demonstrated a RDS phenotype and a delay in DNA repair, similar to HeLa cells overexpressing miR-335 [25]. RS73 also displayed delays in DNA repair demonstrated by γ-H2AX foci kinetics and a G2/M checkpoint defect-a checkpoint that is also dependent on CtIP [28], [38]. In addition, RS73 LCLs were found to have a defect in BRCA1 foci formation [28], similar to miR-335 overexpressing HeLa cells, and subsequent studies of RS7 LCLs also showed reduced BRCA1 foci formation (Figure S6). Taken together, these data support an operational model in which miR-335 impacts the DDR through CtIP and BRCA1. To test this hypothesis, the constitutively miR-335 overexpressing RS7 and RS73 LCLs were treated with AMO-miR-335, which resulted in the partial restoration of BRCA1 foci (Figure 6A). The DDR defects observed in RS7 and RS73 suggest that miR-335 modulates the DDR, and when strongly overexpressed, reduces DNA repair. It also provides one possible explanation for the reduced colony survival observed in these RS-LCLs [22], [42]. To directly demonstrate the connection between miR-335 overexpression and radiosensitivity, we treated RS7 and RS73 cells with AMO-miR-335 and found that clonogenic survival after 1 Gy of IR significantly improved with AMO-miR-335 treatment but was not affected by AMO-Control (AMO-CTL) treatments (Figure 6B). In addition, MCF7 cells have high levels of miR-335 expression and treatment with AMO-miR-335 increased CtIP protein levels (Figure 5C). Pre-IR treatment of MCF7 cells with AMO-miR-335 increased the IR-induced formation of BRCA1 foci at 8 hours and also enhanced the clonogenic survival of cells at different doses of irradiation (Figure S7A and S7B).

## Discussion

We have identified a novel ATM-mediated DDR pathway involving a CREB–miR-335–CtIP axis. The proposed pathway impacts upon BRCA1 foci formation and BRCA1 retention at DSBs, cell cycle checkpoint regulation, DNA repair, and cell survival. This regulatory pathway is responsive to DSBs and our working model places ATM kinase, and the inferred subsequent phosphorylation of CREB, as a novel down-regulating mechanism of *MEST*/miR-335 which supplements the suppression of maternally-derived transcripts by promoter hypermethylation (Figure 1) [29], [30], [36]. The methylation status of the *MEST/miR-335* promoter region remained largely unchanged after IR, indicating that methylation does not play a significant role in the IR-induced modulation of miR-335 (Figure S1B and S1C). The ChIP assay showed that CREB binds directly to the promoter region of *MEST/miR-335* (Figure 2G) and CREB siRNA significantly reduced basal miR-335 expression (Figure 2F), suggesting that CREB is responsible for a substantial portion of the basal miR-335 expression (Figure 2E). This made it technically difficult to fully assess the IR-induced response in cells where CREB was already knocked down. Previous studies indicate that ATM phosphorylates CREB after IR and this phosphorylation reduces the transcription activity of CREB [36]. Our data demonstrating the removal of CREB from the MEST/miR-335 promoter region by IR suggests that CREB is likely responsible for the IR-induced and ATM-dependent downregulation of miR-335.

Tavazoie et al. discovered that miR-335 expression levels were significantly lower in some metastatic breast cancers and that overexpression of miR-335 suppressed tumor metastasis [29]. Interestingly, the MCF7 breast cancer cell line used in our study strongly expresses miR-335 and has reduced BRCA1 foci levels. BRCA1 mutations are associated with a large increase in the risk of breast cancer and our data suggest that miR-335 overexpression may be another mechanism by which the functions of this cancer-associated gene can be modulated. Our results also indicate that miR-335 overexpressing cells are more sensitive to IR-induced damage and that the significantly lower miR-335 expression found in the study by Tavazoie et al. may potentially render breast cancer cells more resistant to IR. This dual role of miR-335 might further explain why some metastatic breast cancers are more resistant to radiotherapy or chemotherapy. These studies suggest that miR-335 is a promising candidate as a radiosensitizer for breast cancer radiotherapy or chemotherapy if miRNAs could be used to target relevant genes. For example, overexpression of miR-335 should cause tumor cells to become more sensitive to IR or DSB-inducing agents, as observed in this study, and continued overexpression should suppress tumor metastasis and tumor re-initiation.

Previous *in vitro* studies have described CtIP functions in the DDR as promoting DSB end-resection via the MRN complex [32], as well as helping to select the DNA repair mechanism activated after DSBs by promoting HRR via BRCA1 recruitment [37]. Thus, reductions in CtIP protein expression could have far-reaching consequences in the DDR, such as the effects on RDS, BRCA1 foci formation, and other DNA repair defects observed when miR-335 was overexpressed in HeLa or was endogenously overexpressed in RS7 and RS73 LCLs. These cells had reduced CtIP protein levels due to miR-335 overexpression and a cellular phenotype consistent with defects in the CtIP-BRCA1-mediated DDR [25], [28]. Transfection of CtIP to miR-335 overexpressing HeLa cells rescued the BRCA1 foci formation and clonogenic survival in these cells, further solidifying the notion that miR-335 modulates DDR through CtIP (Figure 4C and 4D). Interestingly, miR-335 overexpression also changed cell cycle distribution before IR (Figure S4). Given that CtIP has been previously shown to function in cell cycle regulation [22], [41], [43], it will be interesting to learn whether the miR-335-CtIP-regulated cell cycle affects the choice of DNA repair between non-homologous end joining and HRR. Our working model suggests that the downregulation of miR-335 by ATM–CREB in WT-LCLs could result in an increase in CtIP protein expression after IR-induced damage and subsequently enhance the DDR. However, significant changes in CtIP protein levels after IR were not observed in WT-LCLs, using the methods in this study. This suggests that, in WT-LCLs, the ATM–CREB–miR-335–CtIP axis plays a more subtle role in the DDR and most likely functions to ‘fine tune’ DNA repair.

In contrast, RS7, RS73 and MCF7 cells illustrated the substantial influence that miR-335 can have on the DDR when grossly dis-regulated, such as by strong miR-335 overexpression. RS7 and RS73 have defects in post-IR DNA repair and cell cycle regulation and both, similarly, do not efficiently form or retain BRCA1 foci at the sites of DNA damage [25], [28]. Interestingly, both BRCA1 foci defects and reduced colony survival were responsive to suppression of miR-335 levels by AMO-miR-335. The partial abrogation of the defective DDR cellular phenotypes in these cells and in MCF7 cells (e.g. BRCA1 foci) suggests that miR-335 overexpression is responsible for many of the DDR defects observed in these cells and is an example of the substantial impact miR-335 can have upon the DDR. Human CtIP mutations have been implicated in Seckel and Jawad syndromes and cells from these patients present with hypersensitivity to DNA damage [44]. The present study suggests that miR-335 may be an additional mechanism by which CtIP can be disrupted in disease phenotypes and could present with similar clinical features (i.e., phenocopies) while lacking CtIP mutations.

## Materials and Methods

### Cell culture, miRNA precursors, antisense morpholino oligonucleotides, and irradiation

MCF7 cells were cultured in DMEM media with 10% FBS, 1% streptomycin/penicillin/glutamine (PSG) and 10 ng/ml insulin. HeLa cells were cultured in DMEM with 10% FBS and 1% PSG. Lymphoblastoid cell lines were cultured in RPMI1640 media with 10% FBS and 1% PSG. Precursor miR-335 and pre-miR-CTL were purchased from Applied Biosystems (Foster City, CA). Antisense Morpholino Oligonucleotides (AMO) were custom synthesized based on the pre-miR-335 target sequence and conjugated with non-peptide chemicals that are used to deliver AMO-miR-335 intra-cellularly, i.e. vivo-AMOs (Gene-Tools, Philomath, OR). The sequence of AMO-miR-335 is 5′-ATCAACAGATATAAACAGCAGG. A standard control vivo-AMO (AMO-CTL) was also purchased from Gene-Tools. CtIP without the 3′UTR was amplified by PCR against the cDNA generated from HeLa total RNA and cloned into a pcDNA3 vector. Transfections for miRNA precursors were done with Lipofectamine RNAiMax and plasmids with Lipofectamine 2000 (Invitrogen, Carlsbad, CA) following the manufacturer's protocol. LCLs were pretreated with AMO-miR-335 (1 µM) or AMO-CTL (1 µM) for 3 days followed by re-treatment and feeding 1 day before collecting cells for experiments. Irradiation was performed using a Mark IV cesium-137 sealed source irradiator at a dose rate of ∼4.5 Gy/min.

### RNA extraction and real-time quantitative PCR (RT–qPCR)

Total RNA from cultured cells was extracted by the mirVana miRNA isolation kit (Applied Biosystems). TaqMan microRNA expression assays (Applied Biosystems) were used to quantitate mature miR-335 expression following the manufacturer's protocol. U6 expression was used as an internal control for miR-335 expression. *CtIP* mRNA levels were measured by RT-qPCR with the TaqMan Gene Expression Assay. *GAPDH* mRNA was used as an internal control for *CtIP* mRNA levels. Detailed protocols for 5-azacytidine treatment and promoter methylation analysis are provided in Text S1.

### Clonogenic survival assay

HeLa cells were transiently transfected with pre-miR-CTL (50 nM) or pre-miR-335 (50 nM) using RNAi Max. After 48 hours, cells were plated at 500 cells/well in 6-well dishes and then incubated for 24 hours. Cells were then treated with a series of IR doses (0, 1, 2 and 5 Gy) and grown for 10–14 days before staining with 1% crystal violet. Plates were imaged with a VersaDoc Imaging System and clumps of cells containing more than 50 cells were scored as colonies with the Quantity One program (Bio-Rad, Hercules, CA). To generate a radiation survival curve, the surviving fraction at each radiation dose was normalized to that of the non-IR control. The CSA for LCLs was performed as previously described after treatment with AMO-CTRL or AMO-miR-335 [23].

### BrdU incorporation assay

To analyze the S-phase checkpoint, HeLa cells were transiently transfected with pre-miR-CTL (50 nM) or pre-miR-335 (50 nM) using RNAi Max and irradiated with 10 Gy 48 hours after transfection. BrdU was added to cells after a 2 h post-IR incubation to allow S-phase arrest and incubated 2 h for BrdU labeling. Cells were collected by trypsinization and centrifugation. Cells were stained with a FITC labeled anti-BrdU antibody following the manufacturer's protocol for BrdU Flow Kits (BD Pharmingen, NJ) and analyzed by FACS.

### Neutral comet assay (NCA)

The NCA was performed according to the manufacturer's protocol (Trevigen Inc.) and the comet scoring and calculation was done as previously described [26]. Briefly, HeLa cells were transiently transfected with pre-miR-CTL (50 nM) or pre-miR-335 (50 nM) and irradiated with 15 Gy 48 h after transfection. Cells were embedded in agarose gel, lysed *in situ*, electrophoresed, and stained with SYBR Gold. Slides were imaged using an Olympus fluorescent microscope equipped with AxioVision camera and acquisition software. The images acquired were analyzed with Comet Score software (TriTek Corp.) and scored with the Comet Score software as previously described [26]. A minimum of 50 cells was analyzed per experiment and experiments were performed in triplicate. The Tail Moment (TM) was used as the metric to measure DNA damage and the ratio of non-IR and 5 h post-IR samples were calculated to give the “percent DNA repair”. The percent DNA repair was normalized to the WT control. This normalization set the WT control to 100%, or full repair.

### BRCA1-irradiation induced foci (IRIF)

Immunofluorescent detection of IRIF was performed as previously described [45]. LCLs were irradiated with 12 Gy and BRCA1 antibodies were used at 1∶300 (Novus, CO) 8 h post-IR. Following primary antibody incubation, the cells were washed 3 times in 0.1% Triton-X-100 in PBS and blocked again in 10% FBS at room temperature for 1 hr. Coverslips were incubated with 1∶400 Alexa Fluor Anti-Mouse 488 (Invitrogen, Eugene, OR) for 1 hr. After washing 4 times with PBS, coverslips were mounted onto slides in Vectashield with DAPI. Cells were visualized using an Olympus fluorescent microscope and AxioVision Rel. 4.7 software. A minimum of 100 nuclei was analyzed.

### Luciferase reporter assays

For the *CtIP* 3′UTR reporter assay, the full-length 3′UTR of *CtIP* was PCR amplified from genomic DNA of HeLa cells and ligated into the downstream pRL-CMV of luciferase gene in the vector (Promega, Madison, WI). Del6 *CtIP* 3′UTR was generated with QuickChangeII site directed mutagenesis kit (Stratagene). HEK-293T cells were co-transfected with the indicated reporter vectors and pre-miR-335 (50 nM) or pre-miR-CTL (50 nM). A *firefly* luciferase pGL3-control (Promega) was also co-transfected to normalize the transfection efficiency. Luciferase activity was measured with the Dual-Glo™ assay (Promega). Results were expressed as a normalized ratio of *Renilla* to *firefly* luciferase.

### Western blotting

Nuclear lysates from HeLa cells or LCLs were isolated with the NE-PER kit (Pierce, Rockford, IL). Cells were irradiated with 10 Gy, harvested 2 hrs post-IR and then lysed. Equal amounts of total protein for each sample (50 µg or 100 µg) were loaded onto SDS-PAGE and immunoblot analysis was performed with the following antibodies: rabbit-anti-CTIP (Bethyl Laboratory Inc.TX), mouse-anti-beta-actin (Santa Cruze Biotechnology, CA).

### Chromatin immunoprecipitation assay

The Chromatin immunoprecipitation (ChIP) assay was performed using the SimpleChIP Enzymatic Chromatin IP Kit (Cell Signal Technologies, Boston, MA) according to manufacturers procedures. Briefly, ∼4×10∧7 HeLa cells were irradiated or mock irradiated and incubated for 2 hours. Proteins were cross-linked to chromatin 2 hours post-IR and the cells were collected. DNA was fragmented using enzymatic digestion and collected after cell lysis. The digested chromatin was precipitated using an anti-CREB or anti-IgG antibody (Cell Signal Technologies) and purified. Purified DNA was amplified using primers specific to the MEST promoter region (forward: TGTAAAGGAAACCTGCCCCG, reverse: GTGGGTACTGAACCGTGAGA) which yield a product of 232 base pairs. Samples were run on a 2% agarose gel to visualize PCR fragments.

### Statistics

All experiments were repeated independently three times (n = 3) unless otherwise noted. The Student's t-test was used to evaluate the difference of two groups of data in all the pertinent experiments. P-values of <0.05 (using the two-tailed, unpaired t-test) were considered significant. Data is presented as the mean +/− one standard deviation.

## Supporting Information

Figure S1 (A) Demethylation treatment leads to an up-regulation of *MEST* and miR-335. RT-qPCR confirms demethylation of the *MEST* promoter with the demethylation agent, 5′-azacytadine, by showing increases in both *MEST* and miR-335 expression in MCF7 and two WT-LCLs. (B) Representative data showing the fraction of *MEST* DNA copies that were hypermethylated before and 2 hours after 10 Gy IR. (C) Representative data showing the fraction of *MEST* DNA copies that were unmethylated before and 2 hours after 10 Gy IR. No significant change in *MEST* methylation was observed with IR.
(PDF)

Figure S2 RT-qPCR results confirming >100 fold increases in miR-335 expression levels after transfection of pre-miR-335 into HeLa cells.
(PDF)

Figure S3 Western blotting shows down-regulation of CtIP protein expression after miR-335 induction by 5′-azacytadine treatment in 2 WT-LCLs and MCF7 cells, indicating that demethylation of *MEST* promoter leads to reduced CtIP protein expression.
(PDF)

Figure S4 A representative analysis of IR-induced cell cycle intra-S phase checkpoint by FACS. HeLa cells overexpressing miR-CTL or miR-335 were treated with or without 10 Gy IR. DNA synthesis at S-phase was labeled by BrdU. Three independent experiments have been done and summarized in Figure 4A.
(PDF)

Figure S5 Representative comet tail images showed miR-335 overexpression in HeLa cells leads to a delay in DNA repair. Three independent experiments have been performed and summarized in Figure 4B.
(PDF)

Figure S6 RS7 has a BRCA1-IRIF formation defect. (A) BRCA1-IRIFs were assessed 8 hours after 12 Gy IR in a WT-LCL (WT2). RS67 is RNF168−/− and does not form stable BRCA1-IRIFs after IR; it was used as a negative control [27]. RS7 displayed a significant reduction in BRCA1-IRIFs. Cells were scored as positive if they contained >4 foci/nuclei. (B) Representative images of BRCA1 foci for WT, RS67 and RS7 cells 8 h after IR.
(PDF)

Figure S7 MCF7 BRCA1 foci are restored after AMO-miR-335 treatment. (A) BRCA1 IRIF assay 8 hours post 12 Gy in MCF7 cells that have been treated with AMO-CTL or AMO-miR-335. AMO-miR-335 abrogated the BRCA1 foci defect observed in MCF7 cells. ‘*’ indicates p<0.05. (B) AMO-miR-335 treatment also increases the clonogenical survival fraction of MCF7 cells at different doses of IR. ‘*’ indicates p<0.05.
(PDF)

Table S1 Experiment-verified gene targets for miR-335.
(PDF)

Text S1 Methods for 5-azacytadine treatment and promoter methylation analysis.
(PDF)
