# Supplementary figures and images for: ATM–Dependent MiR-335 Targets CtIP and Modulates the DNA Damage Response
Source: PLoS Genet. 2013 May 16;9(5):e1003505. doi: 10.1371/journal.pgen.1003505 (PMC3656122; doi:10.1371/journal.pgen.1003505)

Supplementary Fig.S1:

A.

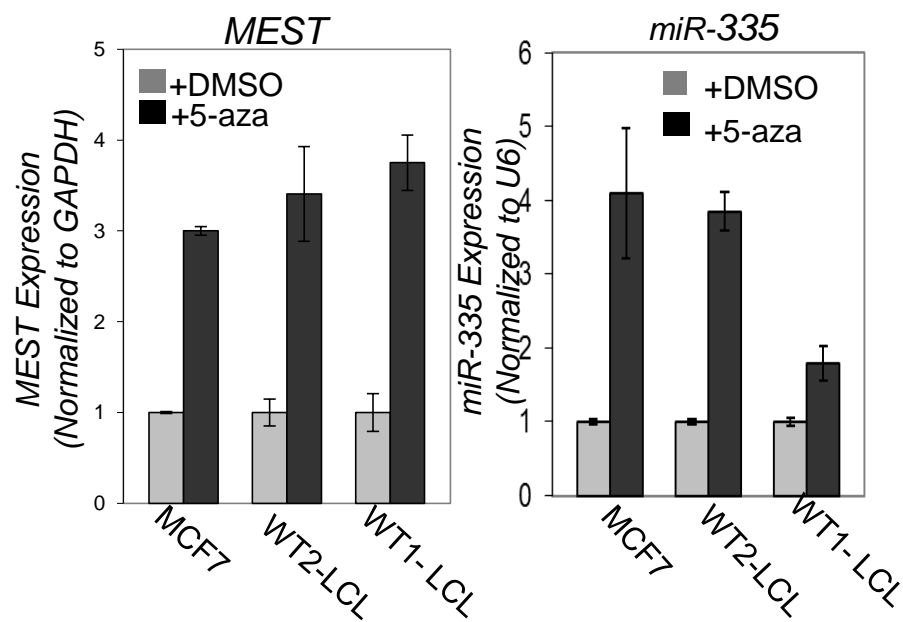

B.

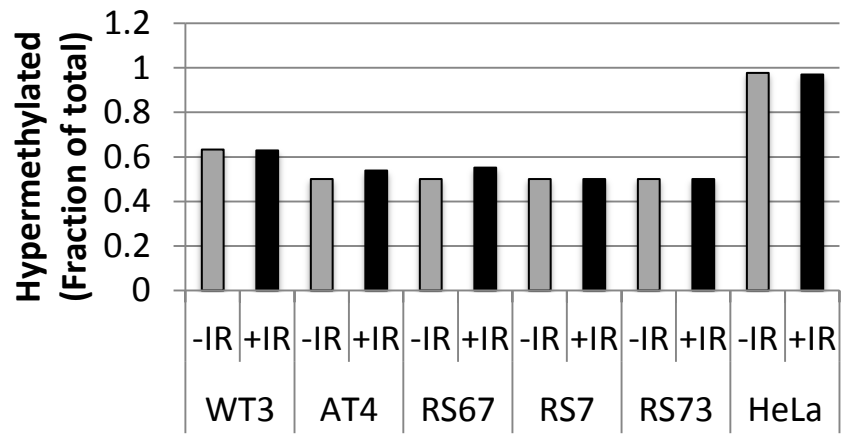

C.

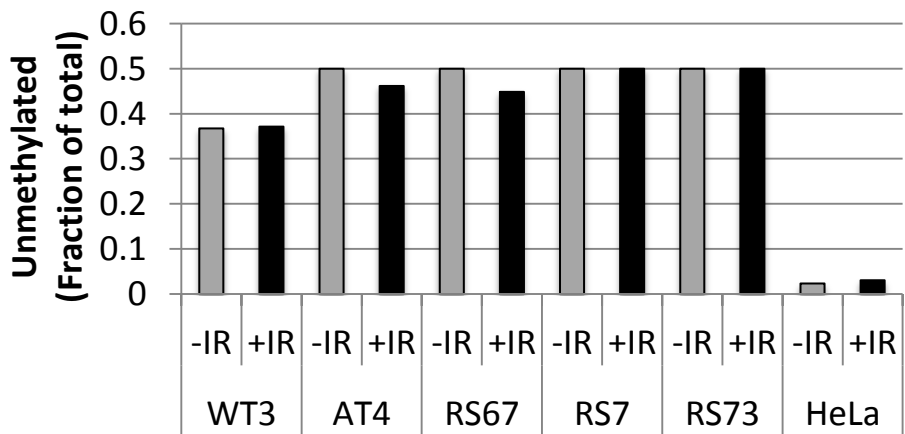

Supplement: Figure S1 — (A) Demethylation treatment leads to an up-regulation of MEST and miR-335. RT-qPCR confirms demethylation of the MEST promoter with the demethylation agent, 5′-azacytadine, by showing increases in both MEST and miR-335 expression in MCF7 and two WT-LCLs. (B) Representative data showing the fraction of MEST DNA copies that were hypermethylated before and 2 hours after 10 Gy IR. (C) Representative data showing the fraction of MEST DNA copies that were unmethylated before and 2 hours after 10 Gy IR. No significant change in MEST methylation was observed with IR. (PDF) [file pgen.1003505.s001.pdf]

Supplementary Fig. S2

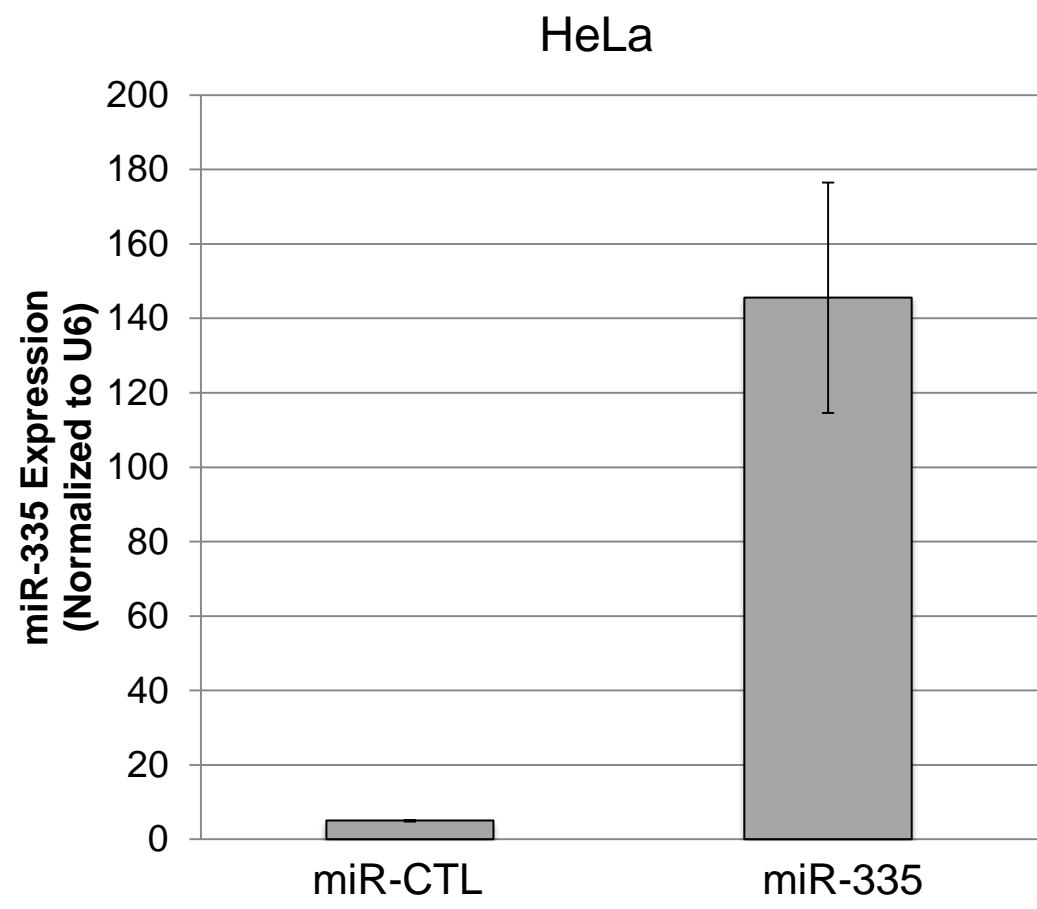

Supplement: Figure S2 — RT-qPCR results confirming >100 fold increases in miR-335 expression levels after transfection of pre-miR-335 into HeLa cells. (PDF) [file pgen.1003505.s002.pdf]

## Supplementary Fig. S3

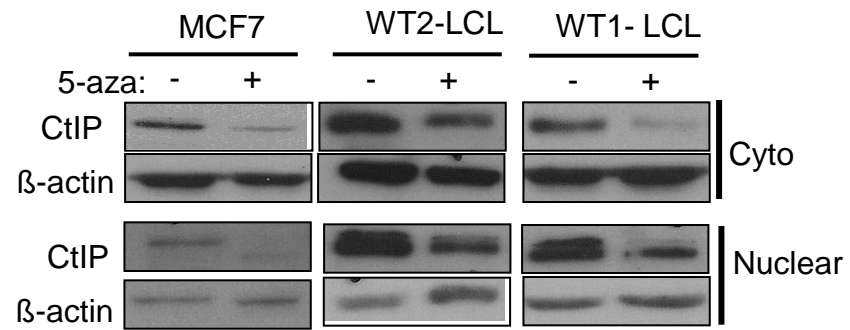

Supplement: Figure S3 — Western blotting shows down-regulation of CtIP protein expression after miR-335 induction by 5′-azacytadine treatment in 2 WT-LCLs and MCF7 cells, indicating that demethylation of MEST promoter leads to reduced CtIP protein expression. (PDF) [file pgen.1003505.s003.pdf]

Supplementary Fig. S4

miR-CTL

Pre-IR

Post-IR

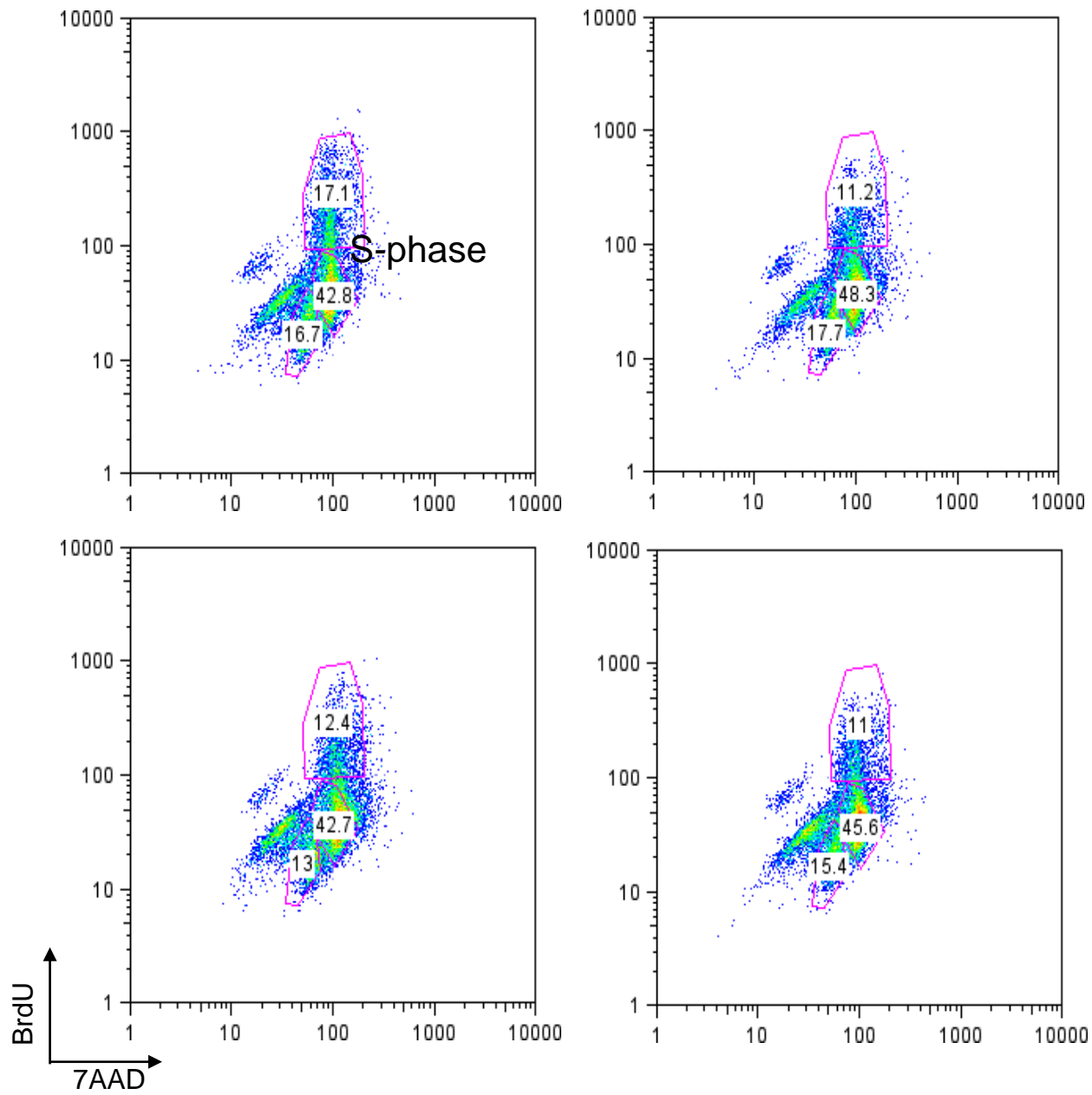

Supplement: Figure S4 — A representative analysis of IR-induced cell cycle intra-S phase checkpoint by FACS. HeLa cells overexpressing miR-CTL or miR-335 were treated with or without 10 Gy IR. DNA synthesis at S-phase was labeled by BrdU. Three independent experiments have been done and summarized in Figure 4A. (PDF) [file pgen.1003505.s004.pdf]

Supplementary Fig. S5

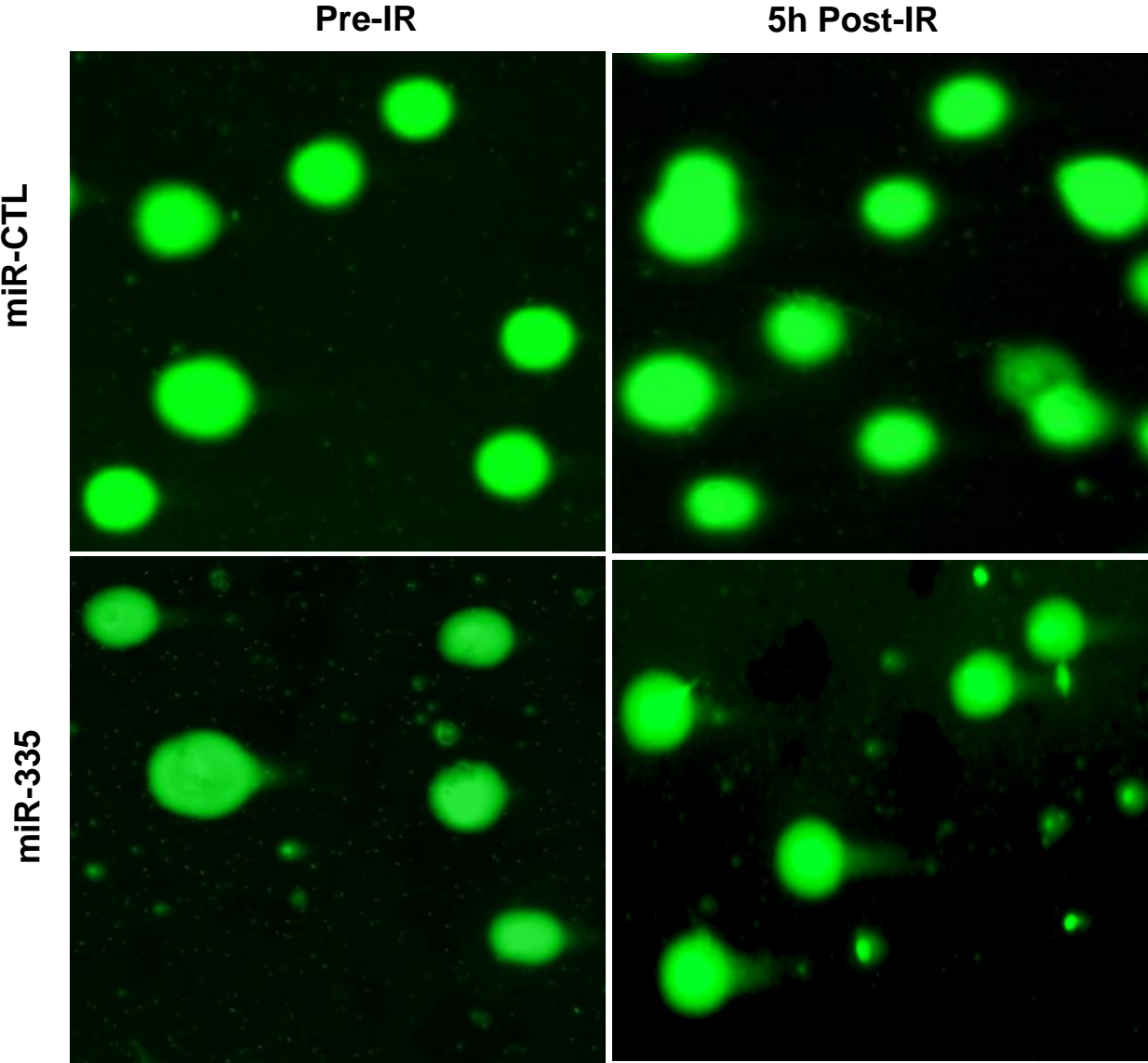

Supplement: Figure S5 — Representative comet tail images showed miR-335 overexpression in HeLa cells leads to a delay in DNA repair. Three independent experiments have been performed and summarized in Figure 4B. (PDF) [file pgen.1003505.s005.pdf]

Supplementary Fig. S6

A.

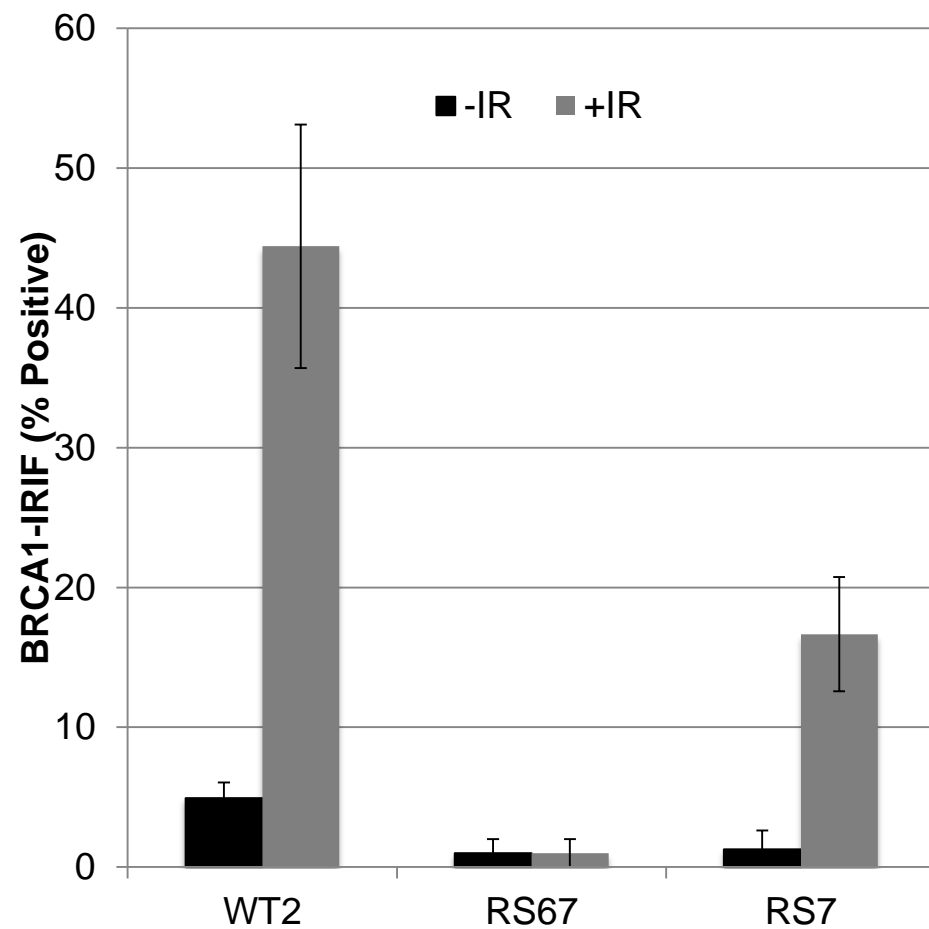

B.

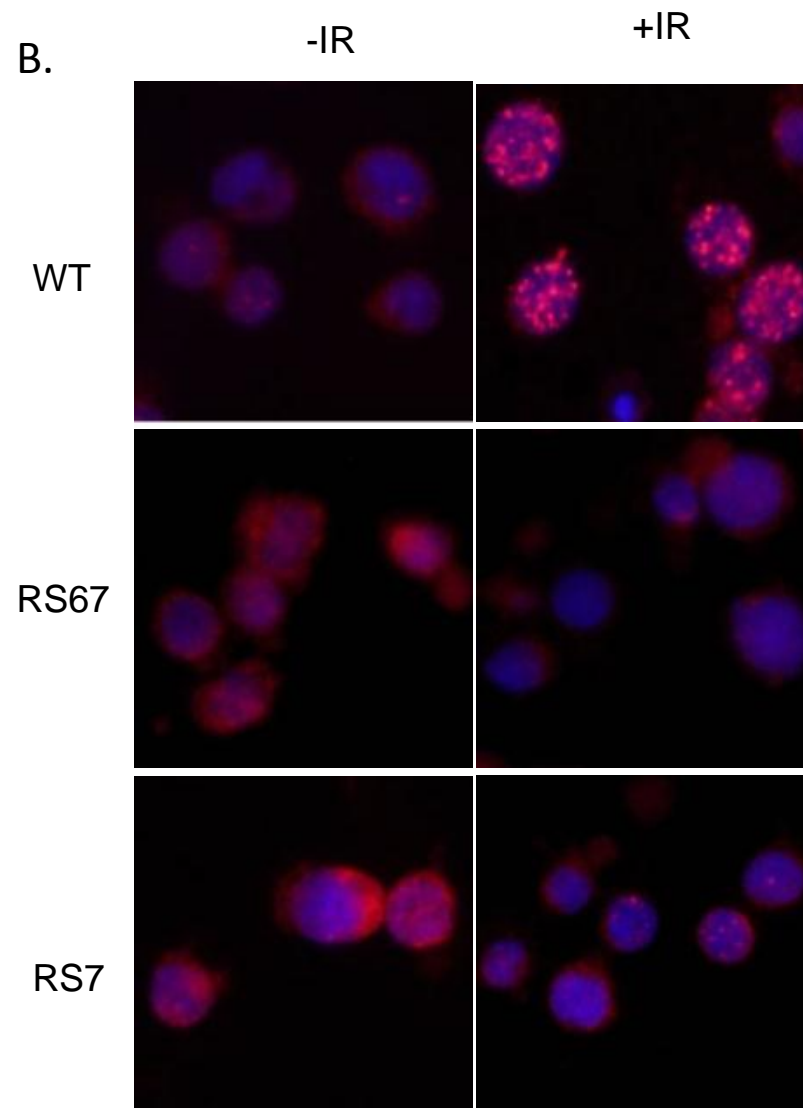

Supplement: Figure S6 — RS7 has a BRCA1-IRIF formation defect. (A) BRCA1-IRIFs were assessed 8 hours after 12 Gy IR in a WT-LCL (WT2). RS67 is RNF168−/− and does not form stable BRCA1-IRIFs after IR; it was used as a negative control [27]. RS7 displayed a significant reduction in BRCA1-IRIFs. Cells were scored as positive if they contained >4 foci/nuclei. (B) Representative images of BRCA1 foci for WT, RS67 and RS7 cells 8 h after IR. (PDF) [file pgen.1003505.s006.pdf]

Supplementary Fig. S7

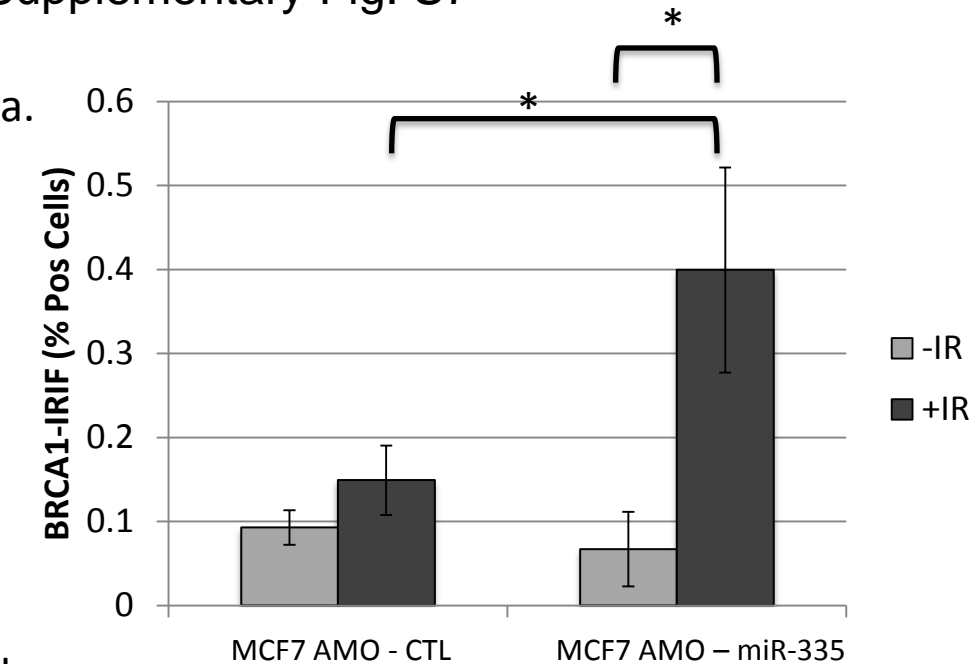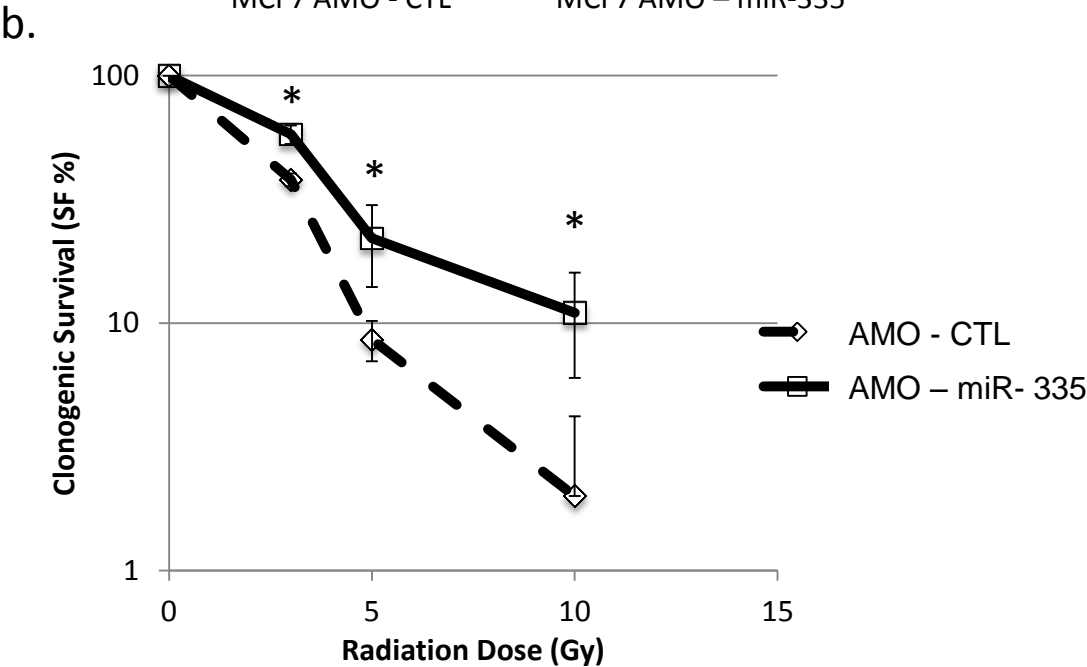

Supplement: Figure S7 — MCF7 BRCA1 foci are restored after AMO-miR-335 treatment. (A) BRCA1 IRIF assay 8 hours post 12 Gy in MCF7 cells that have been treated with AMO-CTL or AMO-miR-335. AMO-miR-335 abrogated the BRCA1 foci defect observed in MCF7 cells. ‘*’ indicates p<0.05. (B) AMO-miR-335 treatment also increases the clonogenical survival fraction of MCF7 cells at different doses of IR. ‘*’ indicates p<0.05. (PDF) [file pgen.1003505.s007.pdf]
