# Supplementary material for: ATM–Dependent MiR-335 Targets CtIP and Modulates the DNA Damage Response
Source: PLoS Genet. 2013 May 16;9(5):e1003505. doi: 10.1371/journal.pgen.1003505 (PMC3656122; doi:10.1371/journal.pgen.1003505)
Supplement: Table S1 — Experiment-verified gene targets for miR-335. (PDF) [file pgen.1003505.s008.pdf]

Supplementary Table 1

Experiment-verified targets for miR-335

Target gene	Function	Reference
SOX4/TNC	Breast cancer metastasis	Tavazoie et al., 2008
Daam1	Astrocytoma cell growth and invasion	Shu et al., 2011
RUNX2	Cell proliferation, migration and differentiation of mesenchymal stem cells	Tome et al., 2011
Bcl-w/SP1	Gastric cancer metastasis	Xu et al., 2012
ROCK1/MAPK1	Neuroblastoma cell invasiveness	Lynch et al., 2012
RB1	Cell proliferation and cell cycle	Shi et al., 2012<br>Scarola et al., 2010
CtIP	DNA damage response	This study

Tavazoie SF et al., Endogenous human microRNAs that suppress breast cancer metastasis. Nature. 2008 , 451:147-52.
Shu M et al., Targeting oncogenic miR-335 inhibits growth and invasion of malignant astrocytoma cells. Mol Cancer. 2011, 10:59.
Tomé M et al., miR-335 orchestrates cell proliferation, migration and differentiation in human mesenchymal stem cells. Cell Death Differ. 2011,18:985-95.
Xu Y et al., MicroRNA-335 acts as a metastasis suppressor in gastric cancer by targeting Bcl-w and specificity protein 1. Oncogene. 2012, 31:1398-407.
Lynch J et al., MiRNA-335 suppresses neuroblastoma cell invasiveness by direct targeting of multiple genes from the non-canonical TGF-β signalling pathway. Carcinogenesis. 2012; 33:976-85..
Shi L et al., miR-335 promotes cell proliferation by directly targeting Rb1 in meningiomas. J Neurooncol. 2012, 110:155-62.
Scarola M et al., miR-335 directly targets Rb1 (pRb/p105) in a proximal connection to p53-dependent stress response. Cancer Res. 2010 ; 70:6925-33.
