# Supplementary material for: ATM–Dependent MiR-335 Targets CtIP and Modulates the DNA Damage Response
Source: PLoS Genet. 2013 May 16;9(5):e1003505. doi: 10.1371/journal.pgen.1003505 (PMC3656122; doi:10.1371/journal.pgen.1003505)
Supplement: Text S1 — Methods for 5-azacytadine treatment and promoter methylation analysis. (PDF) [file pgen.1003505.s009.pdf]

Supplementary Methods:

5'-azacytadine treatment: LCLs or MCF7 cells were treated with 5'-azacytidine (Sigma Aldrich, St. Louis, MO) at 10 μM for 4 days. Cells were collected for total RNA extraction or nuclear and cytoplasmic lysates were isolated as described in 'Western Blotting.'

***MEST* promoter methylation status**

The *MEST* promoter methylation assay was performed using the EpiTech Methyl PCR Assay (Qiagen). 10^7 cells were mock treated or irradiated with 10 Gy and DNA was collected after 2 hours. DNA was processed according the manufactures specifications and amplified using the SybrGreen qPCR assay and MEST primer (Qiagen).
